# The effect of simultaneous exposure on the attention selection and integration of segments and lexical tones by Urdu-Cantonese bilingual speakers

**DOI:** 10.3389/fpsyg.2022.918737

**Published:** 2022-09-07

**Authors:** Jinghong Ning, Gang Peng, Yi Liu, Yingnan Li

**Affiliations:** ^1^Department of Chinese and Bilingual Studies, The Hong Kong Polytechnic University, Kowloon, Hong Kong SAR, China; ^2^Poly U – Peking U Research Centre on Chinese Linguistics, The Hong Kong Polytechnic University, Kowloon, Hong Kong SAR, China

**Keywords:** attention distribution and integration, lexical tones, simultaneous bilinguals, non-optimal perception, phonetic conflicts

## Abstract

In the perceptual learning of lexical tones, an automatic and robust attention-to-phonology system enables native tonal listeners to adapt to acoustically non-optimal speech, such as phonetic conflicts in daily communications. Previous tone research reveals that non-native listeners who do not linguistically employ lexical tones in their mother tongue may find it challenging to attend to the tonal dimension or integrate it with the segmental features. However, it is unknown whether the attentional interference initially caused by a maternal attentional system would continue influencing the non-optimal tone perception for simultaneous bilingual teenagers. From an endpoint in the age of language acquisition, we investigate whether the tone-specific attention mechanism developed by the Urdu-Cantonese simultaneous bilinguals is automatic enough to assist them in adapting to a phonetically-conflicting environment. Three groups of teenagers engaged in a four-condition ABX task: Urdu-Cantonese simultaneous bilinguals, Cantonese native listeners, and Urdu-speaking, late learners of Cantonese. The results showed that although the simultaneous bilinguals could phonologically process Cantonese tones in a Cantonese-like way under a conflict-free listening condition, they still failed in adapting to the phonetic conflicts, especially the segment-induced ones. It thus demonstrated that the simultaneous exposure and years of regular education in Hong Kong local schools still could not automatically guarantee simultaneous bilingual processing of Cantonese tones. In interpreting the findings, it hypothesized that, except for simultaneous exposure, the development of a tone-specific attention mechanism is also likely to be L1-inhibitory, tone experience-driven, and language-specific for simultaneous bilinguals.

## Introduction

The speech variances on an acoustic-to-phonetic level (e.g., sound conflicts, fast-talking speed, talker variances, etc.) may further lead to perceptual barriers for non-native listeners. To deal with the non-optimal conditions, a language-specific selective attention-to-phonology (hereafter, SATP) system can play a vital role for listeners. SATP refers to a sensory mechanism that can cognitively reinforce listeners only to select the language-specific acoustic cues while remaining other redundant and chaotic inputs blend into the background automatically (Francis and Nusbaum, 2002; McCandliss and Yoncheva, 2011; Strange, 2011). In the perceptual learning of a second language (L2), bilinguals, particularly those with reduced proficiency, have to pay efforts to inhibit a SATP transfer shaped by their first language (L1) when perceiving a non-native speech (Trofimovich, 2008; MacWhinney, 2018; Verbeek et al., 2022). The failure to inhibit an L1 transfer on the attentional level may lead to a non-adaptation of the speech variants. Further, such adapting barriers on an acoustic-to-phonetic level may tangle with wrong lexical interpretation from sound to a word and arouse misunderstanding in daily non-optimal listening environments (Bradlow and Alexander, 2007; Meng, 2021; Pelzl, 2021).

In the field of SATP, increasing attention has been paid to the research issue regarding how an early learning age/ early bilingualism modulates bilinguals’ adaptation to the non-optimal listening condition by selecting appropriate L2 cues through the SATP system. The early bilingualism effect on the non-optimal perception of L2 contrasts has been examined by a large number of SATP research, which dominantly focused on segmental or stress learning (Navarra et al., 2005; Dupoux et al., 2008, 2010; Molnar et al., 2014; Hisagi et al., 2015; Yan et al., 2019; Datta et al., 2020). Comparatively, there is a lack of research investigating the early bilingualism effect on SATP about tone learning (e.g., Liu and Ning, 2021). Moreover, it is a research gap about how simultaneous bilingualism modulates bilinguals’ SATP systems in terms of tone perception in a non-optimal condition. In this study, simultaneous bilinguals refer to those who have had extensive exposure to two languages and have continued to use both from the first year of their lives till pre-adolescence (De Houwer, 1990, pp. 3; Sebastián-Gallés et al., 2005). The early sequential bilinguals have fully or partially established an L1-specific SATP before they start learning L2 at an early age (De Houwer, 1990, pp. 2; Meisel, 2004). Comparatively, simultaneous bilinguals are more experienced in utilizing the attentional system to govern and refine two language systems from the very beginning of their childhood. Thus, it is interesting to see if simultaneous bilingualism can guarantee an automatic SATP for bilinguals to adapt to non-optimal and cognitively-demanding environments.

The current study investigates the tone-specific SATP by observing two attentional components under a non-optimal (i.e., phonetic conflicting) condition: attention integration and distribution across linguistic cues (Repp and Lin, 1990; Braun and Johnson, 2011; Lin and Francis, 2014; Zou et al., 2017). Moreover, the automaticity of SATP was examined based on listeners’ accuracy and reaction time (RT) in response to stimuli (Zou et al., 2017; Verbeek et al., 2022). As an experimental group, we recruited simultaneous bilinguals in Urdu (non-tonal) and Cantonese (tonal language). The simultaneous bilinguals were among the second-generation immigrant population in Hong Kong. They had been simultaneously exposed to Cantonese and Urdu through their bilingual parents since they were born in Hong Kong. They kept learning Cantonese regularly after enrolling in local kindergarten and primary schools.

Moreover, they were secondary-school students in Hong Kong at the time of the experiment. Based on participants’ ethnic identity and language proficiency, Urdu was defined as their native language, whereas Cantonese was classified as their non-native language in this study. It is challenging to recruit Urdu/ Cantonese monolinguals in Hong Kong, a dense multilingual city. Hence, we recruited the following two control groups: the native Cantonese teenagers who were dominant in Cantonese and the Urdu native teenagers who were late and low-proficiency learners of Cantonese. All the groups participated in a multi-conditioned ABX task, with or without phonetic conflicts.

## Literature review

### Perceptual barriers in non-optimal listening conditions and the role of bilinguals’ SATP

A typical scenario in our daily communications is that the speeches we perceive are rich in acoustic variations (Crandell and Smaldino, 2000). For example, listening with phonetic conflicts (Bradlow and Alexander, 2007), fast speaking speed (Dupoux et al., 2008, 2010), or other non-optimal conditions involving attention switching from one phonetic dimension to another (Costa et al., 2009; Kalashnikova et al., 2021), etc. Bilinguals may encounter significant barriers in adapting to the above non-optimal listening conditions, even though they may be able to cope with a conflict-free and cognitively-less-demanding condition (Dupoux et al., 2008, 2010; Liu and Ning, 2021). This is also true for tone learning. The tone perceptual difficulty driven by a non-optimal listening condition has been consistently reported for language beginners (e.g., Pelzl, 2021), advanced learners (e.g., Zou et al., 2017), and even the early and highly proficient bilinguals (e.g., Liu and Ning, 2021). Moreover, tonal languages (e.g., Cantonese, Mandarin) use pitch frequency (F0) as one of the primary cues of tones to convey lexical meanings in a large-scale lexicon (Whalen and Xu, 1992; Hallé et al., 2004). Thus, the tone perceptual errors induced by a non-optimal condition on an acoustic-to-phonetic level may further lead to misrepresentation of spoken words, which may thus hinder the mutual understanding in our daily communications (Marslen-Wilson and Warren, 1994; Munro, 2008).

To adapt to a non-optimal speech, bilinguals must develop the ability to select linguistic cues automatically and accurately with the help of a matured SATP system (Lin and Francis, 2014; Zou et al., 2017). According to the Automatic Selective Perception model (hereafter, ASP model; Strange and Shafer, 2008; Strange, 2011), bilinguals’ SATP system may function as a “navigator” to weight, integrate, and map the chaotic inputs to different phonology categories (Francis and Nusbaum, 2002; Ellis, 2006). In this process, how listeners “select/ distribute” (i.e., be sensitive to specific linguistic cues) and “integrate” (i.e., be sensitive to the relation between linguistic cues), linguistic cues are determinant in sharpening their adaptation to a phonetically-conflicting condition (Repp and Lin, 1990; Braun and Johnson, 2011; Lin and Francis, 2014; Zou et al., 2017). In other words, bilinguals’ perceptual barriers in a non-optimal condition may be underlyingly interfered with by their failure to select and integrate different cues on an acoustic-to-phonetic level.

Moreover, the development of bilinguals’ SATP system tends to be a language-specific, L1-inhibitory, and experience-driven process. When establishing an L2 phonology system, bilinguals’ L1-shaped SATP will act as a lens for weighting the perceptual salience of L2 sub-syllabic features (Ellis, 2006; Figueiredo and Da Silva, 2009; Verbeek et al., 2022). With the accumulation of L2 learning experiences, bilinguals can gradually shift their attention to sub-syllabic features from those relevant to L1 to those appropriate to L2 (Kohl, 1993; Steinhauer et al., 2009; Steinhauer, 2014; White et al., 2017). For example, to adapt to speech conflicts, L1 tonal listeners can process incongruent cues accurately and rapidly by selecting and integrating both segmental and tonal dimensions (Wood, 1974; Tong et al., 2008). On the contrary, L1 non-tonal listeners are unlikely to focus on tonal information when perceiving tonal contrasts. Thus, they would find it not difficult to cope with tone-induced conflicts since tones are not salient to contrast meaning in their L1 vocabulary (Braun and Johnson, 2011; Zou et al., 2017). Furthermore, in addition to L1 inhibition and L2 experiences, it has been documented that the automaticity of bilinguals’ SATP system also can be altered by their language acquisition age.

### Effect of early bilingualism on the development of L2-specific SATP

The first year of language exposure is critical for developing bilinguals’ adaptation to phonetic variances (Kalashnikova et al., 2021, see Hendry et al., 2016 for a review). Children’s attention system for a native language is established in the first year of life (Kuhl et al., 1992; Kalashnikova et al., 2021). Early bilingual children, who simultaneously or sequentially acquire a second language, will refine their bilingual phonology and attention system during childhood (Hazan and Barrett, 2000; Verbeek et al., 2022). For specific phonology contrasts, the refining may continue past puberty (9–11 years old) until adulthood (Figueiredo and Da Silva, 2009; Shafer et al., 2011; Yan et al., 2019; Dollmann et al., 2020). In this developmental trajectory, there is an increasing concern about whether the early acquisition age can guarantee bilinguals adapting to a non-optimal condition in L2. To answer this issue, many neural and behavioral studies have examined whether an early acquisition age can prevent bilinguals from suffering a transfer from their L1-specific SATP patterns in a long-term fashion. These SATP-oriented studies usually adopted cognitively-demanding or phonetically-conflicting perceptual tasks (e.g., a task with phonetic incongruence, a speeded identification task, etc.). Most of the above research focused on segmental learning (e.g., Navarra et al., 2005; Molnar et al., 2014; Hisagi et al., 2015; Yan et al., 2019; Datta et al., 2020). Only a few empirical studies examined stress (e.g., Dupoux et al., 2008, 2010) or tone learning (e.g., Zou et al., 2017; Liu and Ning, 2021).

The views are diverse across the above SATP-oriented studies. On the one hand, it was reported that early bilinguals have a far greater cost in attentional resources than monolinguals when adapting to the chaotic L2 cues that are commonly difficult to perceive by non-native listeners (e.g., acoustically non-distinct contrasts or contrasts that are absent from L1). This attentional difference between early bilinguals and monolinguals has been detected both for simultaneous (e.g., stress learning for adults: Dupoux et al., 2010) and early sequential (stress learning for adults: Dupoux et al., 2008; vowel learning for adults: Hisagi et al., 2015; vowel learning for adults: Navarra et al., 2005; vowel learning for infants and bilinguals aged between 3 and 47 years old: Yan et al., 2019) bilingual groups. The above finding may be primarily because bilinguals’ attention device has to simultaneously modulate and navigate two phonological systems (Kalashnikova et al., 2021), usually with reduced experience in each language (Byers-Heinlein and Fennell, 2014). Early bilinguals should be considered a unique population different from monolingual groups (Pallier et al., 2001; Antoniou et al., 2012). On the other hand, some studies utilize easy-to-detect phonetic contrasts (e.g., acoustically distinct, familiar in L1). These studies did not detect a long-term attentional inhibition from L1 for both simultaneous (e.g., vowel learning for adults: Molnar et al., 2014) and sequential (vowel learning for adults and children aged between 9 and 11 years old: Datta et al., 2020; vowel learning for adults: Molnar et al., 2014) early bilinguals. The above finding could be explained by the fact that L2 learning experiences can gradually modulate learners’ L2-specific SATP system (Kohl, 1993; Steinhauer et al., 2009; Steinhauer, 2014; White et al., 2017).

In addition, a little different from the above results in the segment and stress-focused studies, attention-selecting barriers in non-optimal conditions have been detected on the tone level, even for the easy-to-acquire tone contrasts. The acquisition age effect has been examined by tone-specific SATP research for both early sequential bilinguals (e.g., tone learning for middle school-aged children: Liu and Ning, 2021) and late bilinguals (e.g., tone learning for adults: Zou et al., 2017). For example, developed from Zou et al.’s (2017) research design, Liu and Ning (2021) investigated how the early sequential Urdu-Cantonese bilingual teenagers redistributed their attention when selecting segments and tones in phonetically-conflicting conditions. The results revealed that even the early sequential bilinguals fluent in both languages might be confused by the phonetic conflicts. The sequential bilinguals failed to distribute their attention to tones as automatically as tonal native listeners did. Moreover, Liu and Ning (2021) also found that language dominance could positively modulate bilinguals’ tone-specific SATP.

As extension research of Liu and Ning (2021), the current study investigated the tone-specific SATP developed by simultaneous bilingual teenagers. The participants in the current study were employed among Pakistani ethnic immigrants in Hong Kong secondary schools.

### Cantonese lexical tones and early Urdu-Cantonese bilinguals in Hong Kong

Cantonese was chosen as the target tonal language for this study, and it is the most widely spoken language in Hong Kong (Census and Statistics Department of HKSAR, 2021). There are six lexical tones in Cantonese (checked tones are not included): T1 (high-level, pitch value: 55), T2 (high-rising, 25), T3 (mid-level, 33), T4 (low-falling, 21), T5 (low-rising, 23), and T6 (low-level, 22; e.g., Mok et al., 2013). For example, /fu/ means “man” in T1, “caress” in T2, “trousers” in T3, “support” in T4, “woman” in T5, and “attach” in T6 (see Wong and Arai, 2020). Cantonese uses F0 as one of the primary cues to distinguish lexical tone contrasts (Whalen and Xu, 1992; Hallé et al., 2004). Among Cantonese tones, T2 and T4 are acoustically distinct differing average F0, F0 onset, F0 endpoint as well as a contrast pitch direction, with a sharp linguistic boundary between the two categories (Francis et al., 2003; Qin and Mok, 2011; Mok et al., 2018). Urdu, a non-tonal member of the Indo-Aryan family, is Pakistan’s official language (Akkharasena, 2018). There are lexical stress and sentential/phrasal intonation in Urdu, but no lexical tones (Abbasi et al., 2017). A rising contour (LH) is prevalent among speakers as a phrase boundary in Urdu (Jabeen, 2019). Besides, a downward pitch contour (L H L-L%) is predominantly used in Urdu declarative sentences (Jabeen and Hussain, 2012).

Moreover, given that listeners’ SATP may be further integrated with the acquisition difficulty in phonetic learning (García and Froud, 2018; Verbeek et al., 2022), it is also necessary to clarify how Urdu listeners are influenced by language typology when processing Cantonese tones. For example, when perceiving Mandarin tones, L1 non-tonal (English) listeners tend to categorize the mid-rising tone and the high-falling tone in Mandarin into English intonation categories of “question” and “statement” (So and Best, 2011). It deducts that the Urdu listeners may categorize Cantonese T2 as Urdu question intonation due to their similar rising pitch contours. Meanwhile, T4 may be assimilated as downward declarative intonation in Urdu. Therefore, a positive transfer from L1 prosodic typology is predicted for Urdu listeners, implying a relatively easy perception in T2–T4.

Hong Kong is a cosmopolitan city with a large immigrant population in East Asia. According to population reports conducted by the Census and Statistics Department of HKSAR in 2021, about 8% of residents are non-Chinese speakers, with over 90% being non-tonal L1 speakers. Pakistani is one of the largest groups of Hindi-Urdu (non-tonal) speakers in Hong Kong, accounting for 20% of South-Asian secondary school students (Census and Statistics Department of HKSAR, 2017). Meanwhile, 68% of Pakistani students were second-generation immigrants born in Hong Kong, according to a social survey based on a large population of ethnic minority students in Hong Kong (Cheung and Chou, 2018). Additionally, ethnic minority students may have difficulties perceiving and producing Cantonese tones even under optimal conditions, requiring little attentional effort (Wong and Leung, 2018; Yao et al., 2020). In brief, it is of great practical value to examine tone perception learning for second-generation immigrants in Hong Kong who are native speakers of a non-tonal language (e.g., Urdu).

In sum, SATP plays a vital role in facilitating bilinguals to deal with L2 perceptual barriers in non-optimal listening conditions (e.g., Strange and Shafer, 2008; Strange, 2011). In the research field of early bilingualism effect on SATP development, there is a large number of studies focused on segmental and stress learning (Navarra et al., 2005; Dupoux et al., 2008, 2010; Molnar et al., 2014; Hisagi et al., 2015; Yan et al., 2019; Datta et al., 2020). In contrast, much less attention has been paid to the non-optimal tone perception for early bilinguals (e.g., Liu and Ning, 2021). Moreover, there is a research gap on the tonal level about how simultaneous bilinguals would adapt to non-optimal conditions by developing sensitivity and integrability toward L2 tones. It is noteworthy that, though many segmental and stress-focused studies have examined the early bilingualism effect on SATP, it is still necessary to enrich this field from a perspective of tone learning. This is because, as previously mentioned, early bilinguals might encounter attentional barriers, even when perceiving easy-to-detect tones under a phonetically-conflicting condition (Zou et al., 2017; Liu and Ning, 2021). Such result examined in the tone field is thus much different from that in segmental and stress learning (e.g., Dupoux et al., 2010; Datta et al., 2020). Concerning this, the current study tapped into the research issue about the early bilingual effect on SATP by investigating how simultaneous learning influences SATP in tone perceptual learning. This study was an extension work of Liu and Ning (2021), investigating tone-specific SATP for early sequential bilinguals. Also, this study could enrich the line of studies (Navarra et al., 2005; Molnar et al., 2014; Hisagi et al., 2015; Yan et al., 2019; Datta et al., 2020) by providing new evidence from tone learning for simultaneous bilinguals.

## Current study

This study conducted an exploratory work to investigate whether the simultaneous learning experiences contribute to the SATP system when adapting to phonetically-conflicting conditions by the simultaneous Urdu-Cantonese bilinguals. For this purpose, listeners’ attention performance in tone perception was examined with two critical components of SATP development as introduced previously, namely, attention integration and distribution of segments and tones (Repp and Lin, 1990; Braun and Johnson, 2011; Lin and Francis, 2014; Zou et al., 2017). Thereby addressing two questions:

1.Can the simultaneous bilinguals successfully adapt to segmentally or tonally induced speech conflicts by integrally processing lexical tones and segments in Cantonese?

2.Can the simultaneous bilinguals redistribute selective attention to lexical tones as automatically as Cantonese native listeners?

Cantonese native speakers (hereafter, CN), Urdu-speaking Cantonese late learners (hereafter, LL), as well as simultaneous bilinguals in Urdu (native) and Cantonese (non-native; hereafter, SB) were employed as participants. The ABX test revised from the design of Zou et al. (2017) was adopted including four conditions: segment-and-tone (conflict-free), forced-segment (tonally-conflicting), forced-tone (segmentally-conflicting), and segment-or-tone (forced-selecting) conditions. In line with the first research question, comparing the segmentally-conflicting and tonally-conflicting conditions demonstrated how listeners integrate their selective attention on segments and tones. Moreover, the segment-or-tone condition results highlighted how listeners redistribute segmental and tonal information.

Several predictions were made based on the four-condition design. Firstly, when examining experimental effectiveness, it predicted that the CN and SB groups were bound to show the quickest responses in the segment-and-tone condition since listeners can respond either depending on tones or segments as they will. Secondly, the LL listeners might perform poorly compared to the other two subject groups under the forced-tone scenario, where the segmental incongruence may hinder them in identifying speech. It is worth noting that though the LL listeners may assimilate Cantonese T2 and T4 into Urdu prosodic typology, it does not mean that they may allocate their attention more frequently to tonal dimensions than to segments. Moreover, the low Cantonese proficiency might also prevent the LL listeners from achieving satisfactory performance in the segmentally-conflicting condition. As non-tonal L1 listeners, the SATP is shaped in a segment-depend way by the LL group (Braun and Johnson, 2011; Zou et al., 2017). Thirdly, it may not be easy to predict the performance of simultaneous bilinguals precisely. According to Liu and Ning (2021), the early Urdu-Cantonese sequential bilinguals showed a weak performance in the attention distribution task. The early bilinguals thus exhibited an apparent divergence from the tonal native listeners in their study. However, compared with the early sequential bilinguals in Liu and Ning (2021), the SATP device for the SB listeners had to adapt to two sets of language systems from the first year of exposure, which is vital to developing attentional flexibility and adaptation (e.g., Kalashnikova et al., 2021). Hence, it is worth exploring whether language development during the initial years can assist simultaneous bilinguals in acquiring the automaticity of a tone-specific SATP.

The contributions of this study were three-fold: (1) contributing to the ASP model by filling the research gap in tone-specific SATP for simultaneous bilinguals; (2) contributing to bilinguals’ tone learning under a non-optimal condition. Investigating tone-specific SATP would facilitate researchers to better understand how bilinguals deal with perceptual barriers when processing tones in a non-optimal listening environment; (3) contributing to tone learning under the multi-immigrating context in Hong Kong. Since second-generation immigrants (early bilinguals) make up a large population in Hong Kong, this study benefited tone learning for the large group of ethnic minority students in Hong Kong secondary schools.

## Materials and methods

### Participants

A total of 26 Urdu-Cantonese simultaneous bilingual speakers (13 female, 13 male), 27 native Cantonese speakers (14 female, 13 male), as well as 26 Urdu (L1)-speaking late learners of Cantonese (14 female, 12 male) were selected as participants. The SB (mean age = 11.3 years, SD = 1.4) and LL (mean age = 10.8 years, SD = 1.3) participants were Pakistani year-one students in the secondary schools in Hong Kong, where over 50 ~ 80% percent of the students are non-Chinese speakers. The native Cantonese speakers (mean age = 10.7 years, SD = 1.3) were secondary school students in Hong Kong. Due to the Bi-literacy and Trilingualism Language Policy in Hong Kong, all the Cantonese native speakers learned English and Mandarin, but their dominant language was Cantonese. The middle-school-aged students were employed because (1) we needed to compare the performance of middle-school students in Liu and Ning (2021); (2) the period of late childhood (9–11-year-olds) is critical for the refining of the SATP system in distinguishing phonetic contrasts (Figueiredo and Da Silva, 2009; Dollmann et al., 2020); (3) grade one is also known as a typical “transition period” from primary to secondary school for students. At this age, students have an enhanced need to adapt to new and complex speech variances when interacting with unfamiliar teachers and peer groups (Saiegh-Haddad and Geva, 2008; Courtney, 2014). Hence, it had practical values in language education to investigate how middle-school students, who have integrated experiences of simultaneous learning and new transition needs, develop their SATP system to adapt to the non-optimal perceptual environments.

The recommended students and one of their parents received a small compensation gift after completing the online student and parental questionnaires, in either Cantonese or English versions, *via* “google form.” The student questionnaires were designed based on the Bilingual Language Profile (BLP, Birdsong et al., 2012), a widely used tool for assessing speakers’ bilingualism (e.g., Amengual, 2016; Renwick and Nadeu, 2019; Aldrich, 2020). The degree of bilingualism was examined along four modules separately for L1 and L2, including “language history,” “language use,” “language proficiency,” and “language attitudes.” Similarly, the parental questionnaires also consisted of four modules, including “language history for parents,” “language education history for children,” “language proficiency for parents,” and “Urdu proficiency for children.” Also, their Chinese teachers were invited to assess their students’ proficiency in Cantonese. Teachers, students, and parents were required to evaluate on a seven-point Likert scale, with “1” representing “the lowest level” and “7” meaning “the greatest level” for each module except for the “language history.” Moreover, the Pakistani students’ language proficiency was examined by averaging Likert scores rated by teachers/ parents and the students.

According to the parental reports, all parents (N = 102) were non-Chinese residents, with 97.1% of fathers and mothers being Urdu native speakers. 100% of parents agreed that Hindi-Urdu is their children’s native language. Further, we classified the Pakistani students into the LL and SB groups based on their language history, Cantonese proficiency, and the frequency of Cantonese use. Most LL participants in the control group were first-generation immigrants of Hong Kong, born in Pakistan. The LL participants’ parents rarely talked with their children in Cantonese at home, and the LL students were not largely exposed to Cantonese until they were about 7.15 years old (*SD* = 0.92). For language use, the LL students consistently used Urdu across social spheres (e.g., at home, at school, and outside school) with an average use frequency of 6.03 (*SD* = 0.36) in Urdu, which was much higher than their Cantonese use (mean = 1.42, *SD* = 0.42; *t*-test of language use between Cantonese and Urdu: *t*(25) = 32.58, *p* < 0.001). For “language proficiency,” the LL students got a fairly low proficiency score in Cantonese (mean = 2.22, *SD* = 0.26) across language abilities of speaking, reading, writing, and listening, which was far lower than that in Urdu (mean = 5.67, *SD* = 0.42; *t*-test of language use between Cantonese and Urdu: *t*(25) = 16.71, *p* < 0.001). Comparing with the SB group, the LL students got significantly lower scores in Cantonese proficiency (*t*(25) = 28.68, *p* < 0.001) and Cantonese use frequency (*t*(25) = 17.42, *p* < 0.001).

The SB students were all second-generation immigrants born in Hong Kong. Moreover, at least one SB parent was proficient in Cantonese, with a parental rating of 6.1 (*SD* = 0.8) for Cantonese and 6.3 (*SD* = 1.1) for Urdu. Since their children’s infancy, the parents have used Urdu and Cantonese for communication. The pairwise *t*-test suggested no statistical difference in the age of onset exposure (in year) to Urdu (mean = 0.21, *SD* = 0.08) and Cantonese (mean = 0.23, *SD* = 0.07) for the SB students (Table 1, Q i-1). However, before the 3 years old of the child, the parents tended to spend more time using Urdu to communicate with their children than using Cantonese (Table 1, Q ii-1). According to the students’ reports, all the bilingual students had enrolled in local kindergartens and primary schools before entering secondary schools in Hong Kong, where the school instruction languages were either Cantonese or English. At the time of the experiment, all the students had been continuously learning Cantonese for 7.1 years (*SD* = 1.39) through regular classes. The length of formal education in Urdu (mean = 2.19, *SD* = 2.78) ranged widely among the SB students and overall was much less than that in Cantonese (Table 1, Q iii-1). The SB students felt comfortable using Urdu at the age of 4 (*SD* = 0.97), which was much earlier than using Cantonese (mean = 9.19, *SD* = 3.35; Table 1, Q iv-1). For “language use,” the SB students were likely to use Urdu more often in the family while speaking Cantonese more with friends (Table 1, Q i-2, Q ii-2, Q-iii-2). For “language proficiency,” the results reported an equal proficiency in Urdu and Cantonese for reading, speaking, and listening comprehension, but the bilingual students tended to get a lower proficiency in Cantonese than in Urdu for writing (Table 1, Q i-3, Q ii-3, Q iii-3, Q iv-3). In addition, the bilingual students self-evaluated that they got more positive attitudes toward using Cantonese than Urdu (Table 1, Q i-4, Q ii-4).

**Table 1:** Main results of parental and students’ questionnaires (revised from Birdsong et al., 2012).

Question module		Mean value (standard deviations)		*T*-test (2-tailed; *df* = 25) Urdu–Cantonese	
		Urdu	Cantonese	*t*	*p*
**LANGUAGE HISTORY (in years)**					
**Parental**					
Q i-1	What age did your child start to expose to the language?	0.21 (0.08)	0.23 (0.07)	−1.54	0.148
Q i-2	How long did your child live in a family where this language is spoken before he/she was 3 years old?	2.75 (0.41)	2.25 (0.69)	4.372	<0.001***
**Students**					
Q i-3	How long have you received a classroom education in this language?	2.19 (2.78)	7.11 (1.39)	−9.109	<0.001***
Q i-4	What age did you start to feel comfortable using this language?	4.00 (0.97)	9.19 (3.35)	−7.446	<0.001***
**LANGUAGE USE (7-Likert scale)**					
Q ii-1	In an average week, how frequently do you use this at home?	6.34 (1.06)	3.58 (1.10)	9.733	<0.001***
Q ii-2	In an average week, how frequently do you use this with friends?	2.46 (1.21)	4.81 (0.80)	−8.149	<0.001***
Q ii-3	In an average week, how frequently do you use this language with teachers and classmates at school?	4.31 (2.05)	6.04 (0.72)	−4.524	<0.001***
**LANGUAGE PROFICIENCY (7-Likert scale)**					
Q iii-1	How well do you speak it?	6.41 (0.58)	5.88 (0.82)	1.629	0.146
Q iii-2	How well do you understand it?	6.44 (0.48)	6.27 (0.81)	1.159	0.257
Q iii-3	How well do you read it?	6.15 (0.55)	5.83 (0.83)	1.722	0.097
Q iii-4	How well do you write it?	5.55 (0.49)	4.72 (0.61)	5.598	<0.001***
**LANGUAGE ATTITUDES (7-Likert scale)**					
Q iv-1	I identify with this culture	5.58 (1.10)	6.50 (0.81)	3.011	0.006**
Q iv-2	I want others to think I am a native speaker of it.	5.65 (0.69)	6.92 (0.27)	−8.935	<0.001***

For “language proficiency,” “language use,” and “language attitude,” participants estimated on 7-point Likert scale, with 1 representing the lowest level and 7 representing the highest level. The results of pairwise *t*-test (2-tails) between Urdu and Cantonese are shown in the right column. Significant codes: 0 ‘***’ 0.001 ‘**’ 0.01.

Also, it is noteworthy that all the SB students had a long L2 learning experience in English (non-tonal) about 7.31 years (*SD* = 1.41), which was comparable to the length of classroom education in Cantonese (mean = 7.12 years, *SD* = 1.39; *t*-test in education length: English and Cantonese: *t*(25) = 1.04, *p* = 0.306). Besides, bilingual students tended to substantially communicate in English, with a much higher self-rating in English use (mean = 6.23, *SD* = 0.59) than in Cantonese use (mean = 4.81, *SD* = 0.44) averagely across social spheres (*t*-test in language use: English and Cantonese: *t*(25) = 10.11, *p* < 0.001). The bilingual students also frequently used English in daily communication but did not speak another tonal language other than Cantonese (which will be discussed in the paper’s final part).

**Figure 1:**
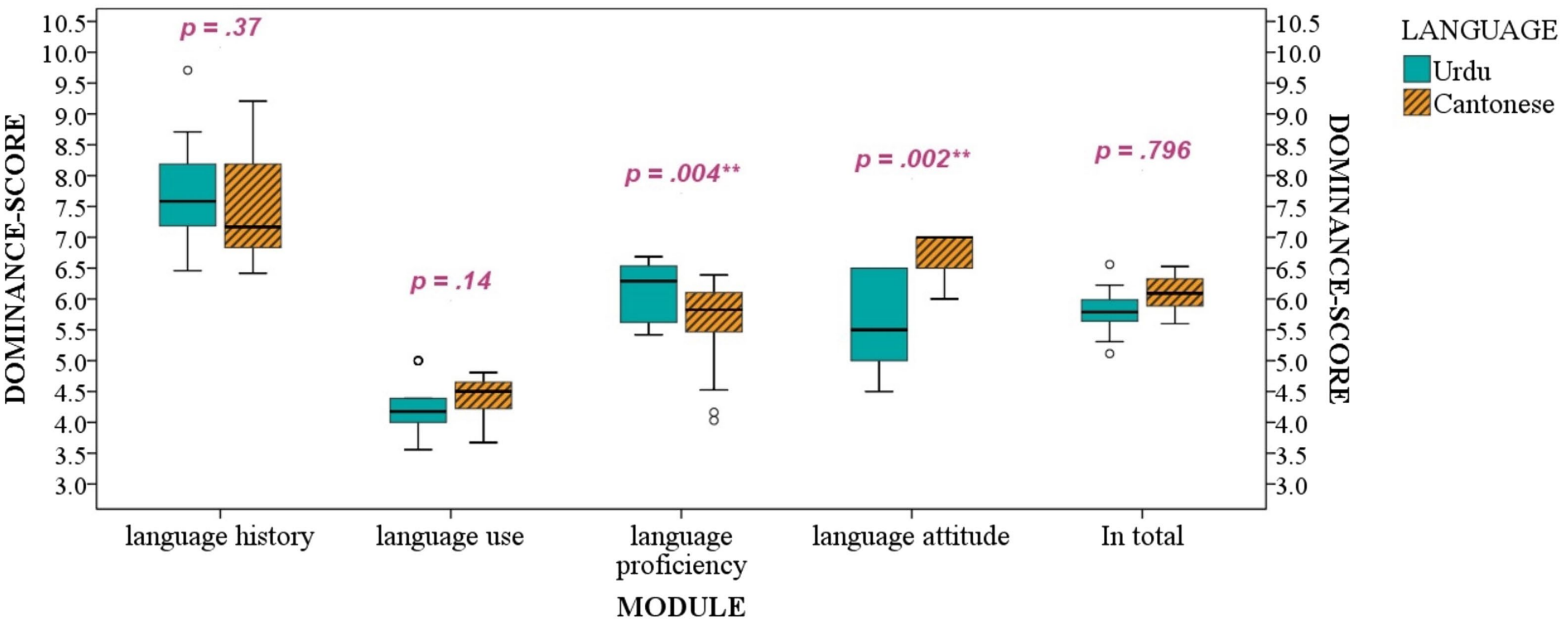
The dominance scores based on BLP calculating method (Birdsong et al., 2012). *T*-test results between Cantonese and Urdu were shown on the chat.

To further examine the dominance degree of Cantonese and Urdu for each bilingual participant, we calculated dominance scores separately for Urdu and Cantonese based on Birdsong et al.’s (2012) method by equally weighting different modules of parental and students’ questionnaires. According to Birdsong et al. (2012), the higher the dominance scores for a specific language, the more likely the bilingual students are to be dominant in that language. The comparison between Urdu and Cantonese in dominance continua shows whether the bilinguals show bias or balance between Urdu and Cantonese. According to Figure 1 and the paired *t*-test results displayed in the figure, the bilinguals showed a balance between Urdu and Cantonese in the modules of “language history” and “language use” since there was no statistical difference between scores of the two languages. In addition, the bilingual students showed a Cantonese bias in the module on “language attitude.” At the same time, they were more Urdu-dominant in “language proficiency.” By equally weighting the estimations across four modules, the total dominance scores showed a balance for the bilingual students using Urdu and Cantonese.

### Stimuli

T2 and T4 in Cantonese were selected as target tonal contrasts. This is because T2 and T4 have acoustically distinct pitch directions (Mok et al., 2013; Chen et al., 2017). As previously predicted, T2 and T4 are easily discerned for Urdu speakers in a conflict-free listening condition (see details in the “Cantonese Lexical Tones and Early Urdu-Cantonese Bilinguals in Hong Kong” section). Thus, adopting T2–T4 could reduce certain negative impedes imposed by Urdu prosodic typology and allow us to concentrate on bilinguals’ attentional performance in segment and tone processing. As shown in Table 2, to avoid lexical interference, two pairs of CVCV disyllabic non-words in Cantonese and Urdu, /kasu/−/tafu/ and /biso/−/diso/, were selected with the target tones on the initial syllables. Disyllables were utilized because the first syllable location can reduce the phonological influence of Urdu sentence-final intonation on bilingual speakers (Zou et al., 2017). The target tone in the first syllable was carried with either Cantonese T2 (high-rising) or T4 (low-falling). The second syllable for each disyllabic non-word was neutralized as Cantonese high-level tones (T1), the most stable tone in Cantonese that can facilitate the discrimination of the adjacent tones (Qin and Mok, 2011). The vowels were [a], [i], [u], [o], and the consonants included [k], [t], [th], [p], [s], [f]. In each non-word pair, the vowels remained unchanged; only the consonant changed in the place of articulation or articulatory manner. Three native Cantonese speakers (two female and one male) were invited to record the disyllables with CoolEdit 2.0 on a Lenovo ThinkCentre desktop computer (i5 core, USB interface: 3.0) with Boom microphone in the audio booth at Hong Kong Polytechnic University.

**Table 2:** Arrangement of stimuli in ABX tasks.

Task	A	B	X
Forced-segment	ka2su1	ta2fu1	ka4su1/ta4fu1
	ka4su1	ta4fu1	ka2su1/ta2fu1
	bi4so1	di4fo1	bi2so1/di2fo1
	bi2so1	di2fo1	bi4so1/di4fo1
Forced-tone	ka2su1	ka4su1	ta2fu1/ta4fu1
	ka4su1	ka2su1	ta2fu1/ta4fu1
	bi4so1	bi2so1	di4fo1/di2fo1
	bi2so1	bi4so1	di4fo1/di2fo1
Segment-and-tone	ka2su1	ta4fu1	ka2su1/ta4fu1
	ka4su1	ta2fu1	ka4su1/ta2fu1
	bi4so1	di2fo1	bi4so1/di2fo1
	bi2so1	di4fo1	bi2so1/di4fo1
Segment-or-tone	ka2su1	ta4fu1	ka4su1/ta2su1
	ka4su1	ta2fu1	ka2su1/ta4fu1
	bi4so1	di2fo1	bi2fo1/di4fo1
	bi2so1	di4fo1	bi4fo1/di2fo1

The number between syllables represents a Cantonese tone mark; 1, 2, and 4 stand for T1, T2, and T4 in Cantonese, respectively. For the stimuli X, “ka4su1/ta4fu1” means X can be “ka4su1” or “ta4fu1” following the A and B stimuli and so are the other X stimuli shown in the table.

ABX test was adopted as the experimental method, with four conditions: Segment-and-tone: participants were required to identify target X that matched in both segment and tonal dimensions; forced-segment and forced-tone: participants were forced to classify the target X along the segmental or tonal dimension, respectively; segment-or-tone: target X matched with either the segmental dimension in A, or tonal dimension in B, and vice versa. The distribution of attention could therefore be observed from the results. The target X contains the same tone or (and) segment as A or B, and the stimuli order can be ABX or BAX. Thus, we got 16 ABX stimuli (two non-word pairs × two Cantonese tones × two AB orders × two matches with A or B). The arrangement of stimuli is displayed in Table 2, which only shows one AB order.

**Figure 2:**
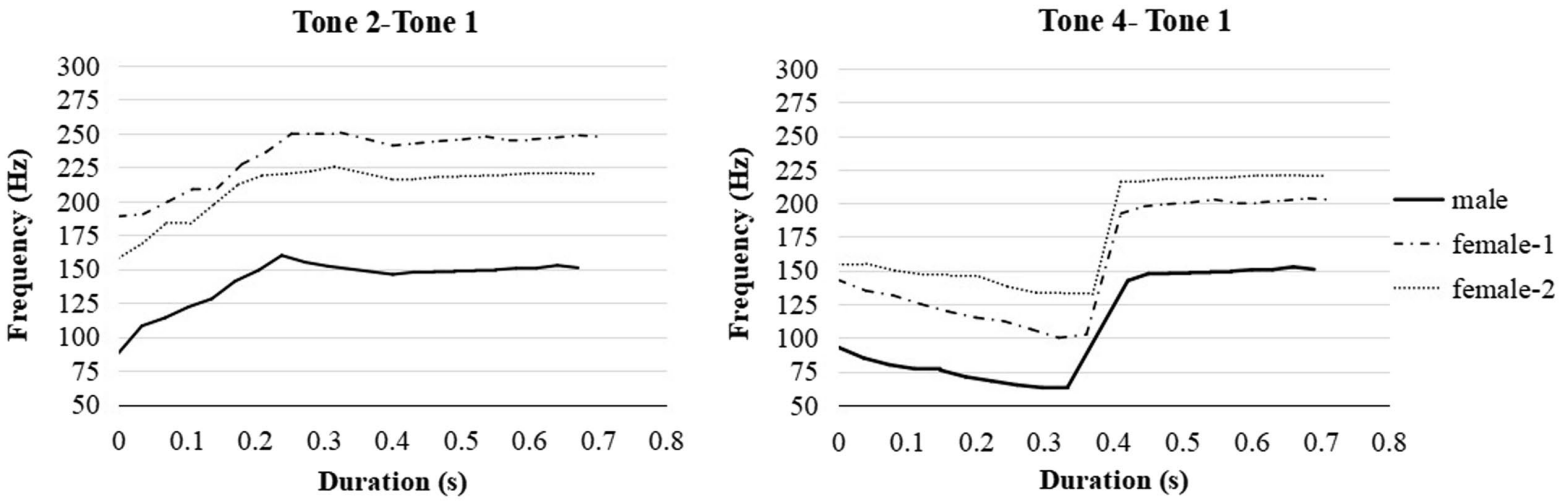
The pitch contours of disyllabic non-words produced by one male native speaker and two female native speakers in Cantonese. The pitch frequencies are averaged across /kasu/−/tafu/ and /biso/−/diso/.

For stimuli recording, three Cantonese native speakers were shuffled in ABX combination instead of being produced by the same speaker (e.g., speaker 1: A, speaker 2: B, speaker 3: X, and vice versa) to avoid the possibility that listeners only process the stimuli acoustically other than referring to the linguistic features (Zou et al., 2017). The non-words were provided in Roman script and Cantonese tone marks (see Table 2). The native speakers, who were previously trained, were asked to produce the disyllabic pairs with an interval of around 1 s in a natural speaking speed. The recordings were saved as 16-bit .wav files with a sampling rate of 44,100 Hz. Figure 2 depicts the averaged pitch contours across stimuli for each speaker. In the first syllable, T2 raised from a low pitch to a higher pitch for each native speaker (female 1: 188 Hz to 250 Hz; female 2: 158 Hz to 225 Hz; male: 88 Hz to 155 Hz). T4 in the first syllable showed a falling contour for each speaker (female 1: 143 Hz to 100 Hz; female 2: 155 Hz to 132 Hz; male: 93 Hz to 63 Hz). T1 in the second syllable showed stable high pitch contours in all syllables. The pitch features obtained from the current study were in line with the description of Cantonese tones in Chen et al. (2017).

### Procedure

The participants were tested separately by sitting in front of a computer (Lenovo ThinkCentre desktop, i5 core, USB interface: 3.0) in a quiet classroom in Hong Kong secondary schools with high-quality headphones (Philips Fidelio X2). The participants were adequately briefed in Cantonese and English about the task procedure by the professional experimenters, who were proficient Cantonese (L1)-English bilingual speakers. The students knew what to do and were required to concentrate on the overall similarity between sounds. The experiment was conducted through the ExperimentMFC script in Praat software (Boersma and Weenink, 2014).

With a quasi-random approach, the four listening conditions (segment-and-tone, forced-segment, forced-tone, and segment-or-tone) were tested with different blocks, and the stimuli were played randomly within each block. The adoption of a quasi-random design was out of concern for the operability of the experiment. A total of 14 local year-one secondary school students (not included in the CN group) were invited to conduct a pilot study designed with different stimuli orders before the formal implementation of the experiment. Besides, a mini-interview was conducted for each student to collect their opinions after the pilot test. The preliminary assessment showed that if the experiment was completely randomized across conditions and stimuli, the ratio of missing trials for most conditions would yield bias in the following analyses. Only when a quasi-random design was used, the portion of missing trials was within an acceptable range (under 10%) according to the statistical guidance research based on a large data set (Dong and Peng, 2013; Jakobsen et al., 2017). In addition, students in the mini-interviews reported that they might feel confused across trials when the stimuli were completely randomized. So they mostly made random judgments (guesses) instead of focusing on the similarity of sounds. Therefore, compared with a full randomized fashion, a quasi-random approach might be more suitable and feasible for the current task, specifically for middle-school-aged students.

In the experiment, the participants focused on a “fixation” shown on the screen (20 ms), then listened to the three sounds, A, B, and X, and indicated if X sounded more similar to the first or second by clicking the mouse on a “1” or “2” shown on the computer screen. The onscreen buttons (“1” and “2”) would appear directly after the three sounds were finished playing. Each task had a 600 ms interval between standard A and standard B, and X appeared after a 900 ms pause (Braun and Johnson, 2011). We applied a comparatively shorter response key duration than Zou et al. (2017) and Liu and Ning (2021). After the “X” ended, the participant who did not give a response within 1,000 ms would receive a reminder onscreen to hurry up. If the participant failed to respond within 2,200 ms, a “failed” hint was shown and passed quickly to the following stimuli. Three-minute familiarization exercises in the segment-and-tone task were given to the listeners before a formal experiment started. Only when the listeners had successfully passed the exercises could they proceed to the subsequent formal task. In the formal experiment, ABX stimuli were repeated three times, generating 192 ABX trials (16 ABX stimuli × four conditions × three repetitions) for each participant. The whole experiment lasted for about 30–40 min for each participant. RT and response type were recorded for each individual, with RT started recording after playing the “X.”

## Results

Segment or tone-based responses were collected for each individual across conditions. Further, accuracy was calculated for the forced-segment, forced-tone, and segment-and-tone conditions, respectively, based on the frequencies of the segment, tone, and segment/tone responses. Since there is no accurate answer for the segment-or-tone condition, the segment-based response rate was calculated. The RT values in the missing trials were involved in the data pool as 2,200 ms. The RT values, which exceeded 3SD below or above mean scores, were excluded for each subject group and experimental condition. Totally we got, for the CN group, 5,156 responses (192 trials × 27 participants-28 missing) and 5,156 RT (192 trials × 27 participants-28 outliers); for the SB group, 4,925 responses (192 trials × 26 participants-67 missing) and 4,914 RT (192 trials × 26 participants-78 outliers); for the LL group, 4,942 responses (192 trials × 26 participants-50 missing) and 4,922 RT (192 trials × 26 participants-70 outliers).

For RT data, the absolute values of skewness ranged between 0.008 and 2.17, and absolute kurtosis values ranged around 0.095–6.96 across sub-groups. Considering the large sample size (*N* > 1,000 per sub-group) in the current study, RT data was determined to obey a Gaussian distribution based on Kim’s (2013)’ criterion (absolute skewness <2 and absolute kurtosis <7). According to Bates et al. (2015), the linear mixed-effect model was performed in R (R Development Core Team, 2008) using the lme4 package for statistical analysis of RT. Besides, the logistic mixed-effect model was utilized for the binary responses using the same package. According to Baayen et al. (2008), the mixed-effect model is advantageous in processing nested hierarchical data. The *p*-values of the models were obtained with the lemerTest package (Kuznetsova et al., 2017). The determination of intercepts and slopes for random effects was based on comparing different models with the likelihood ratio test proposed in Baayen et al. (2008). Marginal *R*^2^ and conditional *R*^2^ were used to examine the efficiency of the models using the MuMIn package (Bartoń, 2015), which, respectively, measure the variances of fixed or fixed and random effects (Nakagawa and Schielzeth, 2013; Zou et al., 2017). *Post-hoc* multiple comparisons across levels were tested with the Multcomp package (Hothorn et al., 2008). Moreover, *p*-values were corrected with Bonferroni adjustment (if necessary) for *post-hoc* multi-comparisons. For statistical analysis in the “Attention Distribution in Segments and Tones” section, two-proportions *z*-tests were conducted with the Stats package (see more details in Wooditch et al., 2021, pp. 180–192) in base R.

**Figure 3:**
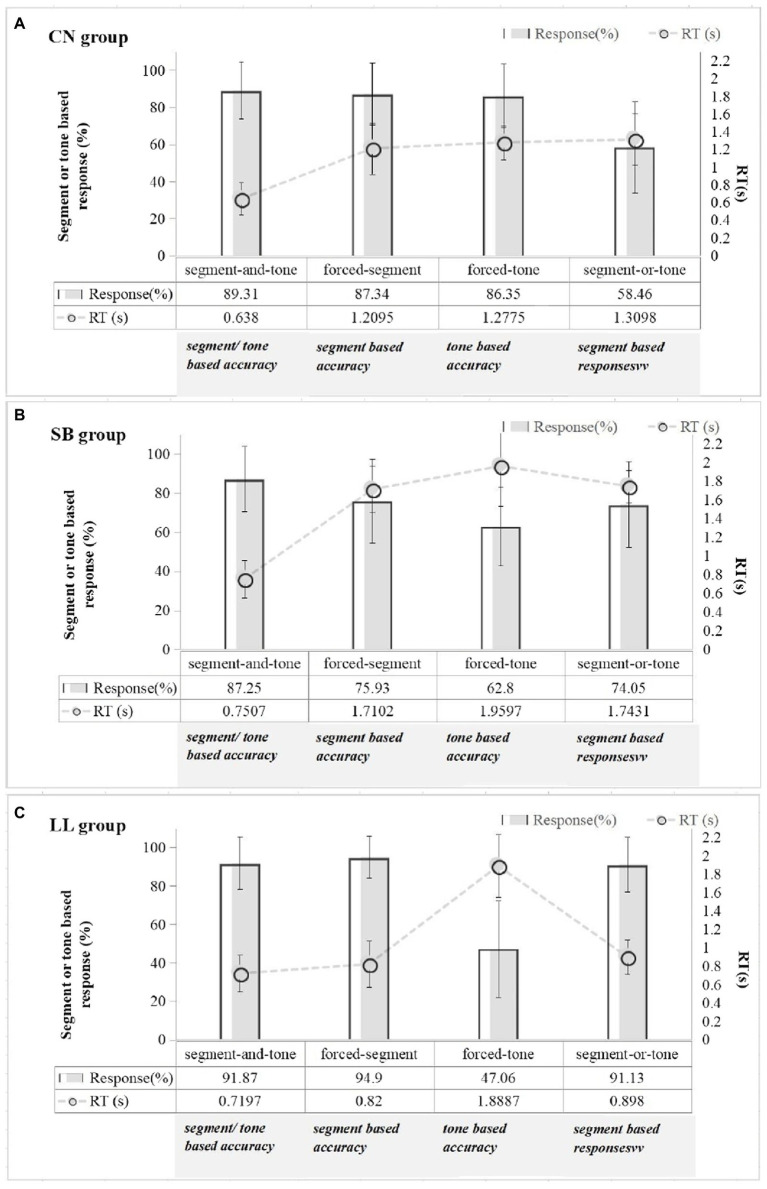
Responses and reaction time for the CN **(A)**, SB **(B)**, and LL **(C)** groups in the forced-segment, forced-tone, segment-and-tone, and segment-or-tone conditions. For the first three conditions, mean values of accuracy were exhibited, and for the last condition, mean response rate in segment was illustrated. For reaction time, 5 times of standard deviation is illustrated with error bar.

Since our research questions should be examined by conducting multiple comparisons across different experimental conditions, different levels of experimental conditions were included in separate models following the research question. In addition, for each model of response and RT, initially, tone type (T2 and T4), consonant type (/b, d, k, t/), and vowel type (/a, i/) were included as fixed effects. However, they were removed from each model because they were not significant as main effects or had no significant interactions with other fixed factors. Figure 3 depicts the average accuracy for the first three conditions, the segment-based response rate for the segment-or-tone condition, and RTs across four experimental conditions for each subject group.

### The effectiveness of the experimental design

In the segment-and-tone condition, the four subject groups achieved high accuracy in syllable classification, with a correct classification averagely ranging above 87% across groups. It ensures that all subject groups could at least robustly rely on one acoustic dimension (segment/tone) to make a decent response to the stimuli. To examine the accuracy in the first three conditions (see Table 3, model 1), a logistic mixed-effect model was finally conducted with the fixed effects of the subject group (CN, SB, and LL), experimental condition (segment-and-tone, forced-segment, forced-tone), and the interaction. Also, the subject and stimuli intercepts were included as random effects. To examine RT values across the four conditions (see Table 3, model 2), a linear mixed-effect model was conducted, with fixed effects of subject group (CN, SB, and LL), experimental condition (segment-and-tone, forced-segment, forced-tone, segment-or-tone), and their interaction.

**Table 3:** The results of logistic and linear mixed-effect models for responses and RT.

Model-1	*glmer(response ~ condition × participate + (1|subject) + (1|stimulus), family = “binomial”)*						
	**Test filed = response**						
	**Fixed effects**	**β**	**SE**	**z value**	**Pr(>|z|)**	**Mar R** ^**2**^	**Con R** ^**2**^
	(Intercept)	0.64771	0.234	2.767	0.00566**	0.551	0.605
	Condition	0.64051	0.091	7.014	<0.001***		
	Participant group	1.313	0.116	11.236	<0.001***		
	Condition: Participant	0.72841	0.044	16.511	<0.001***		
	**Random effects**	**Var.**	**SD**				
	1|subject	0.1261	0.3552				
	1|stimulus	0.1778	0.1333				
Model-2	*lmer(RT ~ L1 × task + (1 | subject) + (1 | stimuli) + (1 | task:stimuli))*						
	**Test filed = RT**						
	**Fixed effects**	**β**	**SE**	**t value**	**Pr(>|z|)**	**Mar R** ^**2**^	**Con R** ^**2**^
	(Intercept)	10.0376	23.6716	23.788	<0.001***	0.811	>0.999
	Condition	10.2312	9.8925	8.095	<0.001***		
	Participant group	3.7547	6.6941	19.711	<0.001***		
	Condition: Participant	12.1318	3.3234	8.0311	<0.001***		
	**Random effects**	**Var.**	**SD**				
	1|task:stimuli	19.9969	6.705				
	1|subject	1.7405	7.602				
	1|stimulus	13.9362	2.3673				
Model-3	*glmer(response ~ condition × participate + (1|subject) + (1|stimulus),family = “binomial”)*						
	**Test filed = response**						
	**Fixed effects**	**β**	**SE**	**t value**	**Pr(>|z|)**	**Mar R** ^**2**^	**Con R** ^**2**^
	(Intercept)	1.54093	0.41672	3.698	0.00021***	0.515	0.673
	Condition	1.38765	0.16057	8.642	<0.001***		
	Participant group	2.83008	0.20015	14.139	<0.001***		
	Condition: Participant	1.25794	0.07542	16.68	<0.001***		
	**Random effects**	**Var.**	**SD**				
	1|subject	5.344	3.468				
	1|stimulus	3.998	3.267				
Model-4	*lmer(RT ~ L1 × task + (1 | subject) + (1 | stimuli) + (1 | task:subject))*						
	**Test filed = RT**						
	**Fixed effects**	**β**	**SE**	**t value**	**Pr(>|z|)**	**Mar R** ^**2**^	**Con R** ^**2**^
	(Intercept)	2.69193	0.13575	134.3001	<0.001***	0.978	>0.999
	Condition	−1.1873	0.06316	134.2994	<0.001***		
	Participant group	−0.5336	0.04516	77.00673	<0.001***		
	Condition: Participant	0.49843	0.02101	77.01085	<0.001***		
	**Random effects**	**Var.**	**SD**				
	1|task:subject	1.1401	0.10782				
	1|subject	15.0245	0.24361				
	1|stimulus	6.1554	0.00212				

Significant codes: 0 ‘***’ 0.001 ‘**’ 0.01.

Moreover, random effects included the subject intercept and stimuli intercept, as well as the by-stimuli slope for conditions. The results indicated that the subject group and experiment conditions had significant main effects and interacted with each other regarding response and RT. It suggests that the attentional resources are consumed differently across conditions and subject groups. In aligning with our predictions, *post-hoc* comparisons were run between the segment-and-tone and the other conditions within each subject group.

CN group (Figure 3A): the CN listeners achieved averagely high accuracy (all mean accuracy > 86%) across the conditions, with no statistical differences between segment-and-tone and the forced-segment (*z* = 1.63; *p* = 0.303), or the segment-and-tone and forced-tone (*z* = 2.32; *p* = 0.093). It suggests that Cantonese native listeners were able to make a correct identification relying on the segments or tones provided in the non-optimal conditions. In the results of RT, the CN group obtained the lowest RT in response to the segment-and-tone condition than to the other three conditions (segment-and-tone and forced-segment: *z* = 31.12, *p* < 0.01; segment-and-tone and forced-tone: *z* = 31.14, *p* < 0.01; segment-and-tone and segment-or-tone: *z* = 31.11, *p* < 0.01). It reflects the fact that the phonetically-conflicting conditions demanded more attentional costs for Cantonese native listeners in comparison with they were in a conflict-free condition.

LL group (Figure 3C): the LL listeners were able to achieve a comparable accuracy in the segment-and-tone and forced-segment conditions (*z* = 1.03, *p* = 0.324). Also, they could process stimuli equally rapidly across conditions except for the forced-tone (RT: segment-and-tone and forced-segment: *z* = 1.09, *p* = 0.237; segment-and-tone and segment-or-tone: *z* = 1.49, *p* = 0.136; segment-or-tone and forced-segment: *z* = 0.13, *p* = 0.891). It may attribute that the segment-and-tone, forced-segment, and segment-or-tone conditions enabled the LL listeners to identify stimuli depending on segmental information. Besides, the LL listeners got much poorer performance in the forced-tone condition compared with what they did in the segment-and-tone condition, with a much lower accuracy (*z* = 21.83, *p* < 0.001) and a much higher RT (*z* = 30.39, *p* < 0.001). It indicates that though the LL listeners could make a quick and accurate response in a conflict-free condition with the help of segmental information, the insufficiency of the learning experience disappointed them when merely tonal information was available in speech.

SB group (Figure 3B): the SB listeners derived a much lower accuracy in the forced-segment and forced-tone than in the segment-and-tone (forced-segment and segment-and-tone: *z* = 7.09; *p* < 0.001; forced-tone and segment-and-tone: *z* = 13.54, *p* < 0.001). In terms of RT, the SB listeners responded fastest in the segment-and-tone compared with what they did in the other three conditions (segment-and-tone and forced-segment: *z* = 30.42, *p* < 0.001; segment-and-tone and forced-tone: *z* = 30.17, *p* < 0.001; segment-and-tone and segment-or-tone: *z* = 30.46, *p* < 0.001). It reflects that the incongruence of tonal and segmental dimensions would largely reduce accuracy and decelerate reaction speed for the simultaneous bilinguals.

The CN and SB groups showed a delayed reaction (long RT) in coping with phonetic conflicts (i.e., forced-tone, forced-segment, segment-or-tone). At the same time, they were responsive when confronting a conflict-free one (i.e., segment-and-tone). Thus, the conflicting conditions required more attentional resources for the CN and SB listeners. In contrast, the LL listeners who were much less sensitive to lexical tones would not feel effortful to neglect the tone-induced conflicts. They thereby would not burn additive attentional resources in the tonally-conflicting conditions such as the forced-segment and segment-or-tone conditions. Besides, the high accuracy obtained in the segment-and-tone condition across subject groups further verified the experimental design’s high effectiveness.

### Attention integration of segments and tones

To examine participants’ performance of attention integration, we compared the forced-tone and forced-segment conditions across the subject groups. A logistic mixed-effect model and a linear mixed-effect model were conducted separately for the response type and RT, with fixed effects of subject group (CN, SB, and LL), experimental condition (forced-segment and forced-tone), and their interaction. The results of the linear and logistic models are exhibited in Table 3 (Model 3 and Model 4). The subject and stimuli intercepts were included as random effects for the two models. Besides, the by-subject slope for the condition was additionally included in the RT model. The results showed significant main effects and interaction of subject group and condition, suggesting that the CN, SB, and LL listeners might have different biases to the segmentally and tonally induced conflicts. *Post-hoc* comparisons were conducted between the forced-segment and forced-tone conditions and between the subject groups.

CN group (Figure 3A): there was no statistical difference in response and RT between the forced-segment and forced-tone conditions (response: *z* = 0.749; *p* = 0.454; RT: *z* = 1.68; *p* = 0.091). However, there was a slight trend that CN listeners got a higher accuracy and shorter RT in the forced-segment condition than in the forced-tone condition.

LL group (Figure 3C): they got a much higher accuracy and a lower RT in the forced-segment condition than in the forced-tone condition (response: *z* = 22.95; *p* < 0.001; RT: *z* = 30.08; *p* < 0.001). It highlights that the mismatch of segmental dimensions would lead to a much more perceptual difficulty for the LL listeners in processing Cantonese tones than the other way around.

SB group (Figure 3B): the SB listeners responded more accurately and quickly in the forced-segment condition than in the forced-tone condition (response: *z* = 13.54, *p* < 0.001; RT: *z* = 30.16; *p* < 0.001). It reveals that the simultaneous bilinguals encountered great difficulty when dealing with the segmental conflicts in tone perception.

CN, SB, and LL groups: for the forced-tone condition, the SB group obtained a far lower accuracy and a longer RT than the CN group (response: *z* = 12.62, *p* < 0.001; RT: *z* = 30.38, *p* < 0.001). But the SB group outperformed the LL group in the forced-tone condition (response: *z* = 7.78, *p* < 0.001; RT: *z* = 21.37, *p* < 0.001). For the forced-segment condition, the SB group exhibited a relatively weak performance compared to the CN (response: *z* = 7.33, *p* < 0.001; RT: *z* = 30.43, *p* < 0.001) and LL groups (response: z = 13.00, *p* < 0.001; RT: z = 30.40, *p* < 0.001).

Combining “The Effectiveness of the Experimental Design” and “Attention Integration of Segments and Tones” sections, it finds that both the CN and SB listeners burned more attentional resources when dealing with a conflicting condition than in a conflict-free environment. The difference is that the CN listeners could still maintain high accuracy when processing conflicting stimuli. In contrast, the SB listeners were disturbed by the phonetic conflicts and thus declined their performance. For the SB listeners, it also illustrates that either tonal or segmental mismatch would hinder their perception of Cantonese speech. The segmental conflicts affected them more than the tonal conflicts. With insufficient Cantonese proficiency, the LL listeners failed in the forced-tone condition, where they could not depend on the segmental information.

### Attention distribution in segments and tones

We compared responses across subject groups in the segment-or-tone condition to investigate how listeners redistribute attention along with segments and tones. The CN listeners attached importance to both the segmental and tonal dimensions in the attention distribution pattern. The LL listeners tended to dominantly rely on the segmental dimension, with a high response rate (above 90%) in the segment. The distribution pattern for the SB listeners was intermediate between the LL and CN listeners, with a 74% chance of attention selection along with segments. According to the repeated two-proportions z-tests, significant distinctions were detected in attention distribution across the LL, CN, and SB listeners: CN and SB: *χ*^2^ = 68.26, *p* < 0.001; CN and LL: *χ*^2^ = 354.71, *p* < 0.001; LL and SB: *χ*^2^ = 125.68, *p* < 0.001.

## Discussion

By referring to the attention integration and distribution of segments and tones, we examined the tone-specific SATP for simultaneous Urdu-Cantonese bilinguals. The results confirmed the high effectiveness of the experiment, with the CN and SB listeners consuming relatively much less RT in the segment-and-tone condition than in the other conditions. It is safe to define the “segment-and-tone” as a low-attention-demanding environment for Cantonese users. The remaining three conditions can be regarded as non-optimal conditions with phonetic conflicts. By further combing the findings of the integration and distribution of segments and tones, the following paragraphs discuss whether the bilinguals’ tone-specific SATP was automatic enough to help them to adapt to the phonetic conflicts. Moreover, we are also concerned about the factors, in addition to simultaneous language exposure, that might potentially alter bilinguals’ attentional performance in the current results.

### The SATP developed by the Cantonese native listeners and late learners

Generally speaking, the CN listeners showed a high cognitive adaptation to the non-optimal environments by maintaining a consistently-high accuracy across the first three conditions. It is thus in line with the ASP hypothesis that native listeners can adapt to a degraded and conflicting listening scenario (Strange and Shafer, 2008; Strange, 2011). For the attention integration, the CN listeners could integrate tonal and segmental information in the conflicting speech since we detected similar performances in the forced-segment and forced-tone for Cantonese native listeners. The performance of the CN group showed that Cantonese native listeners would not ignore both the tonally and segmentally induced phonetic conflicts. This result supports previous studies on Cantonese tone-segment integration (e.g., Repp and Lin, 1990; Schirmer et al., 2005; Lin and Francis, 2014). Also, the result is in line with the results of Braun and Johnson (2011) and Zou et al. (2017), demonstrating that tonal language listeners distribute their attention across tonal and segmental dimensions when processing native speech.

In contrast, the LL listeners only obtained a poor accuracy and a long RT in the forced-tone condition but responded fast in the others. That is to say, the segment-induced conflicts can evoke a perceptual barrier for the LL listeners, while the tonal conflicts only exert limited influence on them. This is mainly because low-proficiency learners may be relatively insensitive to tonal conflicts and have limited exposure to lexical tones. They tend to copy the attention pattern in Urdu by overly relying on segmental information. In addition, the segment-depend distribution pattern detected for the LL listeners echoes that observed for the non-tonal L1 listeners by Zou et al. (2017) and Braun and Johnson (2011).

The CN listeners might have developed a robust and mature SATP specific to Cantonese tones. The LL listeners were sensitive to segmental dimensions, while the CN listeners integrated both aspects of segments and tones. Thus, it is in line with the claims in the ASP model, suggesting that native listeners can select language-specific cues robustly. The finding also supports the view that their mother tongue shapes the “highly over-learned” attention system (Strange and Shafer, 2008; Strange, 2011).

### The SATP developed by simultaneous bilinguals

The SB listeners could make correct and rapid responses in the segment-and-tone condition. However, a noticeable difference was found between the SB and CN listeners when processing in a conflicting environment. In the forced-tone and forced-segment conditions, the SB listeners made far more errors and consumed more attentional resources than the CN listeners. Thus, the SB listeners paid considerable attentional efforts but still struggled to adapt to the phonetic conflicts. It supports the statements in the ASP model. When an optimal condition is provided, non-native listeners are likely to perform well. They have ample time to refer to the L2 knowledge and extract sufficient information to make a correct decision (Strange, 2011).

Notably, the segment-induced and tone-induced conflicts did not decelerate the SB listeners’ performance to the same extent. The SB listeners resulted in much lower accuracy and a far longer RT in the forced-tone condition than in the forced-segment condition. It showed that segmental information was more important for bilinguals when judging tones than vice versa. Hence, it is hypothesized that simultaneous bilinguals might establish a weaker link between tones and segments than Cantonese native listeners will.

Regarding the results of attention distribution, around 74% of stimuli were classified relying on the segmental dimension by the SB listeners, implying that the SB listeners were more sensitive to segmental information than tonal information. Thus, the SB listeners tended to show an intermediate performance between the CN (segment-selection rate = 58%) and LL (segment-selection rate = 91%) listeners in the attention distribution pattern.

Generally, we observed a noticeable reduction in accuracy and a high cost in attentional resources for the SB group in conflict environments. It implied a clear distinction between the SB and CN groups in integrating and redistributing segments and tones. As previously demonstrated, the experimental design showed high effectiveness. The Cantonese tones employed (T2 and T4) are distinct enough to be easily distinguished by non-native listeners. Hence, the differences in performance between the SB and CN groups most likely stem from the SB listeners’ immature development of a tone-specific SATP mechanism. Simultaneous bilinguals cannot be considered “native” in the same way as Cantonese native speakers are. Their non-automatic SATP may thus subsequently influence the integration and selection of tonal information to interpret words and sentences in daily conversations. The results corroborate the statements in Antoniou et al. (2012), illustrating that bilingual speakers should be treated as a unique and configured speaker group from a monolingual one since they have to accommodate their perceptual system to more than one language.

Compared with the results in Liu and Ning (2021), where the SATP detected for the sequential Urdu-Cantonese teenagers lagged behind that of the Cantonese native listeners, the current study further confirmed such attentional divergence between early bilinguals and native listeners. It expanded the claim to include simultaneous bilinguals. This is possible because, with reduced experience in the language (Byers-Heinlein and Fennell, 2014), even early simultaneous and sequential bilinguals’ attention devices may slow down in order to navigate two phonology systems (Shafer et al., 2011; Hisagi et al., 2015; Kalashnikova et al., 2021), especially in a non-optimal listening condition (Strange and Shafer, 2008; Strange, 2011).

### To ASP model: Evidence from an endpoint of age in tone acquisition

The current study contributed to the ASP model in two ways. Firstly, it highlighted the fact that SATP is language-specific. Native listeners extract L1-specific information automatically through complex speech contexts. In contrast, non-native listeners may find it effortful to redistribute and integrate attention to the non-native cues, especially those that cannot be linguistically used in their L1 (Strange and Shafer, 2008; Costa et al., 2009; Strange, 2011; Kalashnikova et al., 2021). Second, it depicted a picture of a tone-specific attention mechanism from the angle of an endpoint in the age of tone acquisition. The simultaneous bilingual students in the current study were exposed to Urdu and Cantonese from the start of their lives through their multilingual parents. Even after years of regular Cantonese instruction from kindergarten to secondary school in Hong Kong, they could not adapt to a phonetically-conflicting condition when perceiving Cantonese tones. In other words, first-year exposure is insufficient to guarantee that simultaneous bilinguals establish a mature attentional mechanism for tone acquisition. It uncovered that, in addition to simultaneous exposure, more factors might be promising to play integrated roles in developing a tone-specific SATP system for simultaneous bilinguals.

#### Interference from an Urdu-specific SATP

ASP model predicts that bilinguals may use L1-specific SATP as a lens for weighting L2 cues (Ellis, 2006; Trofimovich, 2008; Verbeek et al., 2022). Due to the initial attentional transfer from Urdu, bilinguals may be constrained to rely overly on segments. In the current results, the SB listeners showed an intermediate performance between the CN and LL groups regarding attention distribution and integration. Besides, they were more interfered with by segmental violations than tonal ones. Presumably, the SB listeners were suffering from inhibiting an attentional pattern in Urdu when perceiving Cantonese tones. It hypothesizes that the initial SATP transfer will likely affect simultaneous bilinguals as they enter late childhood or pre-adolescence.

#### Individual differences in tone-related experience

According to Liu and Ning’s (2021) findings, there was a greater than 70% chance that the early sequential bilinguals who were dominant in Urdu (L1) used segments to distinguish Cantonese (L2) speech. While more than 60% for the Cantonese-dominant bilinguals. As shown previously, the current simultaneous bilinguals were estimated as balanced language users of Urdu and Cantonese. They allocated the same amount of attention to segments (segment-selection was around 74%) as the above Urdu dominants. As can be seen, except for simultaneous exposure, language use and proficiency are, therefore, of extreme importance to the development of tone-specific SATP (Steinhauer et al., 2009; Steinhauer, 2014; White et al., 2017). In the current study, the bilingual dominance of the simultaneous bilinguals was calculated based on a relative comparison of Urdu and Cantonese rating scores with the BLP method. However, according to participants’ self-reports, the bilinguals might also use English across social contexts, owning to the multilingualism in Hong Kong society. As previously highlighted, English use (Likert score = 6.23) was even scored higher than the use of Cantonese (Likert score = 4.81) and Urdu (Likert score = 5.14) by the simultaneous bilingual participants (all *p* < 0.001). However, extensive use of English, a non-tonal language, is not directly helpful in enhancing bilinguals’ sensitivity to tonal features. Insufficient tone use may be very likely to prevent simultaneous bilinguals’ from establishing the automaticity of the SATP system.

#### Differences between the Cantonese and Mandarin-specific SATP

According to Zou et al. (2017), the Dutch-speaking advanced late learners of Mandarin obtained an accuracy comparable to Mandarin native listeners when integrating and redistributing segments and tones. However, there was a slight trend for both beginners and advanced learners to exhibit slower responses than native Mandarin speakers. Compared to the advanced late learners in Zou et al. (2017), the simultaneous bilinguals in the current study demonstrated weakened attention integration and distribution patterns. Hence, delayed development in tone-specific SATP is revealed in Cantonese acquisition. According to the studies on tone learning of South-Asian students in Hong Kong (Wong and Leung, 2018; Yao et al., 2020), early Cantonese learners persistently made errors in perceiving and producing tones even in optimal conditions, where an attentional effort was not highly demanded. Thus, the delayed acquisition of Cantonese tones (also mentioned in Yao et al., 2020) will increase the likelihood that initial SAPT transfer will affect tone learning in later stages of life for simultaneous bilinguals.

#### Differences between tone and segment-focused SATP studies

This study utilized the falling and rising tones, which are documented to be easily discriminated by learners under optimal listening conditions (Francis et al., 2003; Qin and Mok, 2011; Mok et al., 2018). Like Liu and Ning (2021) and Zou et al. (2017), the current study suggested that the phonetically-conflicting environment can cause specific perceptual barriers for bilinguals in perceiving lexical tones, even though the target tones are theoretically easier to be acquired by language learners. Differently, as introduced previously, the segment-focused SATP research reported that early bilinguals would show non-adaptation to a phonetically-conflicting condition only when they are distinguishing the difficult-to-detect segmental contrasts (Navarra et al., 2005; Hisagi et al., 2015; Yan et al., 2019), other than the easy-to-detect ones (Molnar et al., 2014; Datta et al., 2020). Hence, there is an apparent discrepancy between the results in the above tone studies and the segment-based SATP studies. One reason is that the different experimental designs (e.g., monosyllabic or disyllabic stimuli, words or non-words, task types, etc.) in tone and segment-focused studies may directly lead to different results. The other reason is that there may be a lag in language learners’ acquisition of tones. For example, by investigating tone acquisition under an optimal condition by ethnic minority children in Hong Kong, Yao et al. (2020) found that tonal errors are more severe than segmental errors in bilinguals’ production and perception. Similarly, in Chen et al.’s (2016) large corpus study, adult learners made much more tone errors than segment errors.

The future study suggests incorporating more pairs of tonal contrasts to examine if there exists an interplay between attentional inhibition and tone typology for bilinguals when accommodating a non-optimal listening condition. For example, in Cantonese, T2–T5 and T3–T6 are difficult-to-detect tone contrasts (e.g., Qin and Mok, 2011; Mok et al., 2018). It is thus interesting to examine whether and how different learners adapt themselves in a non-optimal listening condition when perceiving T2–T4, T2–T5, or T3–T6 contrasts.

## Conclusion

The optimal and non-optimal conditions were provided to listeners to investigate how simultaneous exposure influences bilinguals’ distribution and integration of selective attention when processing Cantonese tones. The results showed that the simultaneous bilinguals could process Cantonese speech accurately and quickly when both segmental and tonal dimensions were provided (i.e., segment-and-tone). However, they were more likely to retain an Urdu-like attentional strategy in processing Cantonese tones, especially when the segmental dimension of the stimuli was mismatched. The current study provides evidence for the ASP model through tone acquisition by simultaneous bilinguals. The current study also hypothesizes that the development of simultaneous bilinguals’ tone-specific attention system could result from various factors, including the individual variances in tone-related experiences, language-specific differences, and L1-inhibition, in addition to an early learning age.

## Data availability statement

The raw data supporting the conclusions of this article will be made available by the authors, without undue reservation.

## Ethics statement

The studies involving human participants were reviewed and approved by the Human Subjects Ethics Subcommittee of the Hong Kong Polytechnic University. Written informed consent to participate in this study was provided by the participants’ legal guardian/next of kin.

## Author contributions

JN, GP, and YL contributed equally to the experiment design, conduction, data analysis, and manuscript drafting. YLN also made a significant contribution to the manuscript drafting and revision. All authors contributed to the article and approved the submitted version.

## Funding

This work was funded by the Language Fund under Research and Development Projects 2018–19 of the Standing Committee on Language Education and Research (SCOLAR), Hong Kong SAR, and the Hong Kong Polytechnic University Projects of 4-88F3 and G-UALY. The work described in this paper was partially supported by a fellowship award from the Research Grants Council of the Hong Kong Special Administrative Region, China (Project No. PolyU/RFS2122-5H01).

## Conflict of interest

The authors declare that the research was conducted without any commercial or financial relationships construed as a potential conflict of interest.

## Publisher’s note

All claims expressed in this article are solely those of the authors and do not necessarily represent those of their affiliated organizations, or those of the publisher, the editors and the reviewers. Any product that may be evaluated in this article, or claim that may be made by its manufacturer, is not guaranteed or endorsed by the publisher.
